# It will be worth it, in the end: a model of naturalistic intertemporal choice

**DOI:** 10.3389/fpsyg.2026.1797154

**Published:** 2026-06-26

**Authors:** Philip Newall, Mike W. Peacey

**Affiliations:** 1 School of Psychological Science, University of Bristol, Bristol, United Kingdom; 2 Experimental Gambling Research Laboratory, School of Health, Medical and Applied Sciences, CQUniversity, Melbourne, VIC, Australia; 3 Applied Psychology, Warwick Manufacturing Group, University of Warwick, Coventry, United Kingdom; 4 Chair of Marketing, Technical University of Munich, Munich, Germany; 5 Behavioural Science Centre, University of Stirling, Stirling, United Kingdom; 6 School of Economics, University of Bristol, Bristol, United Kingdom

**Keywords:** addiction, discounting, intertemporal choice, procrastination, time preference

## Abstract

Intertemporal choices, which involve consequences spread over time, often display a bias toward short-term rewards, which was recognized in ancient Greek philosophy as “akrasia.” This bias is nowadays often modelled via hyperbolic discounting models, where intertemporal choice failings are due to an inherently irrational way of trading-off present and future rewards. Hyperbolic discounting models lead to precommitment as a suggested strategy to avoid short-term rewards, as in the Greek story of Odysseus and the sirens. We contrastingly propose a boundedly rational model of naturalistic intertemporal choice, where the complexity of the choice sets lead to people discounting future rewards rationally, but only up to a finite, mentally simulated horizon. This is consistent with only three unique types of error which we link to the established naturalistic biases of addiction, procrastination, and choosing smaller-sooner over larger-later rewards. A second assumption regarding the attentional resources required to simulate outcomes at varying delays then leads to the well-known phenomenon of “preference reversals” — where these three errors tend to recur in the present despite a stated preference to do better in the future. Importantly, extending mental simulation further into the future reduces all these errors, and allows people to act more in accordance with their true preferences. This prediction is consistent with a nascent empirical literature on successful intertemporal choice interventions, which all appear to succeed by helping people to think about the future. This model’s psychological mechanism can help inform the design of future intertemporal choice interventions.

## 1 Introduction

Everyday life contains many intertemporal choices, which involve consequences spread over time. Dietary choices, for instance, trade short-term pleasure for long-term health. Anyone can freely assign their own values to such trade-offs, yet a common human failing involves striving toward a long-term reward but falling short by caving in to short-term temptations. For example, a dieter might order a healthy salad, but then break their resolve later by ordering a calorific dessert. Similar trade-offs occur with smoking, drinking, procrastination, homework, and undersaving for the future. In all these instances, we often tend to plan for the more rewarding long-term action, but cave in to what feels better in the short-term when it becomes immediately available. Ancient Greek writers such as Aristotle, Plato, and Socrates, called these failing “akrasia” (Ainslie, 2001). We refer to them as “intertemporal choice failings”.

Early work on intertemporal choices came from animal studies (e.g., Chung and Herrnstein, 1967), where animals were presented with choices that led to rewards that varied with delay and size^1^. Ainslie (1975) observed that, like humans, animals tended to prefer a larger reward when choosing among delayed options, while preferring a smaller reward when choosing among more immediate options. This led Ainslie (1975) to propose the theory of “hyperbolic discounting” (explained further below) to explain this inconsistent behavior. Ainslie’s solution to the inconsistency involved making a binding precommitment toward the long-term reward, a behavior that he saw some animals learn in his experiments, and which he also saw in the ancient Greek story of Odysseus and the sirens.

Since them, studies have extended this work to human participants choosing between monetary and other rewards in laboratory-controlled settings (Frederick et al., 2002). While many studies’ results are consistent with the failings described by hyperbolic discounting (Ainslie and Haslam, 1992), many are not. Human choices vary across trials (Dai and Busemeyer, 2014; He et al., 2019), depend on how rewards are described (Read et al., 2005, 2017), and change when rewards are nonmonetary (Cubitt et al., 2018). Some contexts even reverse the usual bias toward short-term rewards (Eil, 2012; Loewenstein and Prelec, 1991). Experimental anomalies may also arise from the complexity of reward streams (Enke et al., 2023). The empirical literature on laboratory intertemporal choice is now vast (Rambaud et al., 2023) and has inspired many competing models (Ballard et al., 2023; Scholten and Read, 2010; Scholten et al., 2024).

Our focus, by contrast is on the naturalistic intertemporal choice failings that were known to ancient Greek writers, and which are still experienced by many people today. These people might be trying to control their weight, save for the future, or stop procrastinating over an important homework assignment. No single simple model is likely to be capable of explaining the wealth of empirical observations across laboratory and naturalistic domains. Artificially generated sequences of rewards that are presented in the context of a laboratory experiment are a very different context to the scenario of a dieter in a restaurant presented with a dessert menu. Given this, we believe that the significant welfare impacts occurring from naturalistic intertemporal choice failings justify our focus on this domain.

Psychologically, our model departs from the most widely-used intertemporal choice model by assuming that people must *mentally simulate* the future when facing novel or important choices (Atance and O'Neill, 2001). Mental simulation is considered a key adaptive advantage for a changing future (Suddendorf and Corballis, 2007). Introducing mental simulation to an intertemporal choice model in effect relaxes an assumption shared by the rational and hyperbolic discounting models, that decision-makers can effortlessly access a mental picture of future events. As will be argued in the section called “Thinking about the future in a changing world”, this is a strong assumption to make in a complex and changing world.

In terms of practical implications, our model’s key benefit is the addition of a new strategy for those who want to act in greater accordance with their long-term goals. While the hyperbolic discounting literature focuses on the importance of precommitting to long-term goals (Ainslie, 1975; Laibson, 1997), precommitment can limit flexibility in changing circumstances. The present model instead predicts that people can act more in accordance with their long-term goals by deliberately thinking further into the future. This is a benefit that the present model also has compared to other descriptive theories of intertemporal choice failings beyond hyperbolic discounting, which is discussed further in Section 4.3 below.

The rest of this work is structured as follows. The rational intertemporal choice model is first described, before we sketch the practical difficulty of putting this model into practice in changeable naturalistic environments. We then outline the biases in naturalistic environments which motivate this work, before their explanation via hyperbolic discounting is described. This description mentions how the hyperbolic discounting model in effect makes the same assumption as the rational model, that decision-makers can effortlessly access a mental picture of future events, and how hyperbolic discounting focuses on precommitment as a cure for these failings. We also describe how other descriptive models of intertemporal choice failings might similarly not empower people with cognitive strategies that they can use to avoid these failings. We then describe the assumptions underlying our mathematical model, how this can lead to the intertemporal choice failings that we seek to explain, and how the errors are reduced by further mental simulation of the future. We then review some complementary prior evidence, before providing some suggestions for empirical testing of this model, before concluding. The present work is theoretical in nature. Our aim is to articulate a framework that organizes existing findings and generates clear and testable predictions (several of which are outlined in Section 7). We hope this framework encourages independent empirical investigation, particularly in naturalistic domains.

Our contribution differs from existing approaches in three main ways. First, we formalize intertemporal choice using a truncated attentional horizon (S), which generates discounting-like behavior without assuming an irrational discount function. Second, we provide a simple taxonomy of error types that map onto common real-world behaviors such as procrastination and addiction. Third, we identify attentional allocation to future simulation as a potential behavioral lever, suggesting a practical strategy for improving a wide array of intertemporal choice failings.

## 2 Rational intertemporal choices

The theory of how to make rational decisions originated in finance, where the objective is to maximize wealth. Financial choices generally involve an interest rate (e.g., the interest rates offered by a bank, or the minimum return that an investor wants to achieve). Importantly, interest requires compounding over time, which can be simplified with the mathematical constant *e* (Maor, 2009), such that:

$$\text{present value}*e^{\left(\text{interest rate}*\text{time}\right)}=\text{future value}$$

Compound interest can also be applied in reverse, to deduce that the amount of money that would have to be deposited today to yield a future amount. E.g. at a 10% yearly interest approximately $0.37 would need to be invested to have $1 in 10 years’ time is:

$$\text{present value}=\text{future value}*e^{\left(-\text{interest rate}*\text{time}\right)}$$

$$\text{\$}0.37=\text{\$}1*e^{\left(-0.1*10\right)}$$

This operation is called *discounting*, and can be calculated by flipping the sign applied to the interest rate in the exponent of *e*. When compounding or discounting, the difference between present and future value grows larger as either the interest rate or the length of time increases.

Simple financial decisions use interest rates to convert all cash flows received over time into a single time period, then and select the investment that has the highest value. Samuelson (1937) repurposed this approach to intertemporal choice, replacing money with utility (a numerical measure of happiness), and interest rates with an individual’s “discount rate” (their subjective rate of time preference). A higher discount rate represents a stronger preference for present utility. Because discount rates reflect individual preferences, there is no intrinsic “correct” choice in a given intertemporal choice problem; only correct choices given individual’s preferences (as summarized by the discount rate).

**Figure 1:**
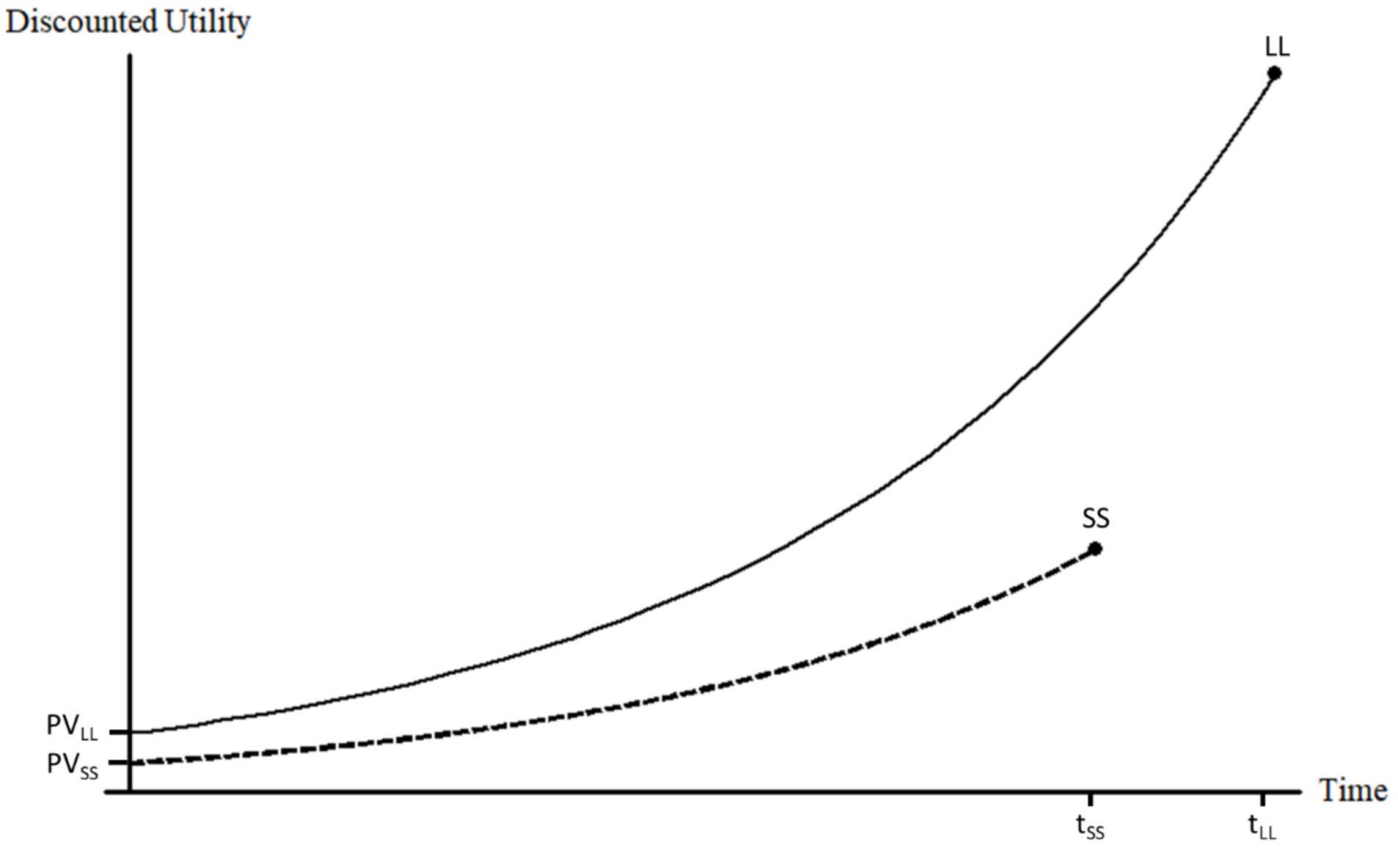
Rational discount curves for two sources of utility occurring in the future. The “smaller-sooner” prospect (SS) occurs at an earlier time (t_SS_) than a “larger-later” (LL, occurring at t_LL_) prospect. The discounted utility of the larger-later prospect, PV_LL_, is higher than the discounted utility of the smaller-sooner prospect PV_SS_, meaning that the larger-later prospect is preferred today. These curves never cross, illustrating consistent preferences over time.

Samuelson knew that using an identical discount rate in each period would ensure that the option that looked best now would continue to look best at all future time periods. This is the feature that makes the rational intertemporal choice model’s choices *consistent*, in that a choice made in the present will be adhered to in all future time periods. The investment which maximizes present value will also maximize the value of the investment at other time periods, for example when the investor expects to retire and start living off the proceeds of their wealth. This means that rational intertemporal choice model discount curves do not cross, as illustrated in Figure 1. However, this result holds only if all sources of future utility are considered, a point which will be returned to in the section called “Three errors.”

## 3 Thinking about the future in a changing world

We use the example of a financial analyst estimating optimal stock market investments to demonstrate an implicit psychological assumption in the rational intertemporal choice model (an assumption shared by the hyperbolic discounting model). Both assume that, no matter what, an individual “knows” their future utilities, at least on average. Although this might hold in a stable and predictable world, real decisions feature many large and unpredictable changes.

Suppose an analyst tracks about 45 firms within one of the 11 major stock market sectors (Kenton, 2019). To evaluate each firm the analyst would have to produce future dividend estimates based on many factors, including each firm’s ability to withstand current and possible future regulatory regimes, global macroeconomic trends, trends in consumer demand, and the value of patents or other protections from competition (Greenwald et al., 2004). When the world changes in some unpredictable way, such as the threat of a trade war, the analyst must rapidly re-evaluate all these estimates. The rational model assumes that these calculations are done instantly and without cost, anytime the world changes. This is a strong assumption, even for an analyst who is expert in her sector, with the benefit of computers and other decision aides. Extending this assumption to all everyday intertemporal choices makes it even less plausible.

Our model replaces this assumption with a psychologically grounded one, that decision makers must first begin by constructing an image of future outcomes before choosing. Based on evidence on how people actually think about the future, we assume that people can harness their attentional resources to mentally pre-experience future events from a personal perspective. This ability has been called episodic future thinking (Atance and O'Neill, 2001). In our framework, individuals evaluate options based on how far they can mentally project into the future, denoted by their subjective attentional horizon, S. Utilities beyond this horizon are effectively ignored. Thus, realized intertemporal choices depend not only on underlying preferences (as in rational and hyperbolic models) but also on the depth of mental simulation. The proposed strategy for mental simulation effectively assumes that we think about intertemporal choices by beginning in the present and gradually simulating into the future. We argue that this assumption reflects the way we do think about the world over time. For example, we try to predict how a film might end by imagining scenarios from the opening scene, and we try to predict a plane’s flight by tracing out its current trajectory, much as we mentalize the movement of an interlocking set of cogs (Hegarty, 2004). In addition to this assumption, there are two reasons why a strategy of limited mental simulation beginning from the present could be considered a boundedly-rational use of scarce cognitive resources.

First, Simon (1955) considers this in the context of bounded rationality when playing chess. Simon suggests that, “[A] player could adopt some kind of planning horizon and include in [her] estimated pay-off the ‘goodness’ of [her] position at the planning horizon.” (p.113). Simon noted this might be a good strategy for intertemporal problems, “If there is time-discounting of pay-offs, this has the advantage of reducing the importance of errors in estimating these depreciated values” (p.113). If the discount rate is positive, then on average long-term utilities will matter the least, and so neglecting these long-term utilities will be less bad than neglecting present or short-term utilities.

Second, if the long-term is more uncertain than the short-term (Gabaix and Laibson, 2017), then it again makes sense to begin simulating intertemporal preferences from the present, which decision makers will be most certain about. In effect, this makes the proposed strategy ecologically rational in a world that deviates from the assumption of perfect forecasting, assuming that uncertainty grows over time.

This approach respects the fact that the world is complex, and that decision makers do not inherently “know” future utilities without thinking about them. When cognitive resources suffice to extend simulation to all relevant outcomes (S = T), the model collapses to the standard rational case. But limited attention or uncertainty shortens the horizon, introducing interesting systematic deviations from rationality. In a changing world, no one can spend endless time predicting every future possibility.

Our model relies on only one other assumption, which drives the fourth intertemporal bias that we seek to explain. This assumption is consistent with other intertemporal choice research and is, we argue, psychologically plausible. We assume that scarce attentional resources are needed to expand S, which prevents the decision-maker from optimally setting S to T for all decisions. We assume that attentional resources are consumed from the first non-zero utility in the choice set, and that it takes fewer attentional resources to expand S over a given time period as that period is moved further into the future. In other words, it takes more attentional resources to expand S over the next month, than it would take to expand S over the same calendar month next year. Section 5.2 below will provide a full set of psychological justifications for this assumption, which includes logarithmic time perception: the observation that one unit of psychological time corresponds to a longer interval of objective time in the long- than short-term (Ventre and Martino, 2022; Ventre et al., 2023; Zauberman et al., 2009). Section 5.2 also shows the behavioral predictions which follow from the addition of this assumption to the model.

## 4 Intertemporal choice failings

The deviations from the rational intertemporal choice model that we aim to help understand all involve a bias toward the short- over the long-term. Now, someone could have a preference for the short-term that is fully-consistent with the rational intertemporal choice model, in that they have a persistent preference to get things now (they have a high discount rate), and they apply this to all future time periods consistently. Our definition of intertemporal choice failings is more specific than this, applying strictly to choices that the individual later comes to regret. Intertemporal choice failings that are later regretted are not compatible with the rational intertemporal choice model, due to the fact that rational intertemporal choices are, as explained earlier, consistent. These are the failings that motivated the creation of hyperbolic discounting models (Frederick et al., 2002).

Before explaining how hyperbolic discounting models work, we will first review four naturalistic intertemporal biases which are consistent with hyperbolic discounting (and not with the rational intertemporal choice model).

### 4.1 Four intertemporal choice biases

- *Addiction* is perhaps the most readily observed intertemporal bias. Addictions involve high initial benefits but significant delayed costs. In addiction research these delayed costs are generally assumed to be so large that the addict, when reflecting upon their situation, would prefer abstinence to the addiction, but fails to achieve this outcome (Orford, 2001). Some research suggests that addiction is associated with mentally simulating events over short time horizons. When asked to complete the story, “After awakening, Bill began to think about his future. In general, he expected to…” heroin addicts responded with an average horizon of nine days, compared to an average horizon of 4.7 years for matched controls (Petry et al., 1998). Although some economists have modelled addiction as a rational consideration of future consequences (Becker and Murphy, 1988), this is not the dominant view in addiction research, which demonstrates the loss of life, health, and wellbeing among addicts and their social circles (Orford et al., 2013). Addiction has featured frequently in the hyperbolic discounting literature (Bickel and Marsch, 2001; MacKillop et al., 2011).
- *Procrastination* may have less noticeable consequences than addiction, but is also highly prevalent, with for example up to 46% of college students in one study nearly or always procrastinating when writing a term paper (Solomon and Rothblum, 1984). We tend to procrastinate, to harmfully delay, activities or purchases with immediate negative consequences, but high overall long-run rewards. This is despite the knowledge that delay is harmful compared to immediate action (Akerlof, 1991; Steel, 2007). Dieting, exercise, and studying can all follow this temporal pattern of utilities. Unlike addiction, with its noticeable consequences, the ultimate loss of human welfare from procrastination is difficult to measure. This could be one reason why procrastination features in the hyperbolic discounting literature (O'Donoghue and Rabin, 1999), but less frequently than addiction.
- The *smaller-sooner bias* involves taking something small now, instead of some sufficiently larger-later reward. For example, a worker might decide to spend her whole month’s pay cheque rather than invest anything for her retirement. This bias is widespread, with half of all American workers in 2016 being estimated to be at risk of not having adequate funds to maintain their lifestyle in retirement (Munnell et al., 2018). The smaller-sooner bias has been frequently illustrated in the intertemporal choice literature with experimental monetary rewards (Frederick et al., 2002). But the smaller-sooner bias appears highly domain-general, and does not just occur with financial decisions.
- *Preference reversals* involve people intending to do better in the future, but then later failing as temptations become presently available, a precise theoretical prediction of hyperbolic discounting (Ainslie, 1975). Preference reversals appear to affect all of biases i.-iii, where these three errors recur in the present despite a stated preference to do better in the future. An addict might intend to get clean *soon*, a procrastinating student might intend to start studying *tomorrow*, and an employee might intend to increase their pension contributions with *next month’s* salary. But when the time comes, these intentions are often not carried out (Schelling, 1984).

### 4.2 Intertemporal choice failings as modeled by hyperbolic discounting

In the rational intertemporal choice model, a single discount rate applies across all time periods. Hyperbolic discounting modifies this by replacing the constant rate with a discount function that declines over time, producing heavier discounting of near-term rewards. This feature means that failings occur in the short-term, even though they are intended to be avoided in the long-term. This can result in a preference reversal (Ainslie, 1975; Laibson, 1997; Loewenstein and Prelec, 1992; Strotz, 1955).

**Figure 2:**
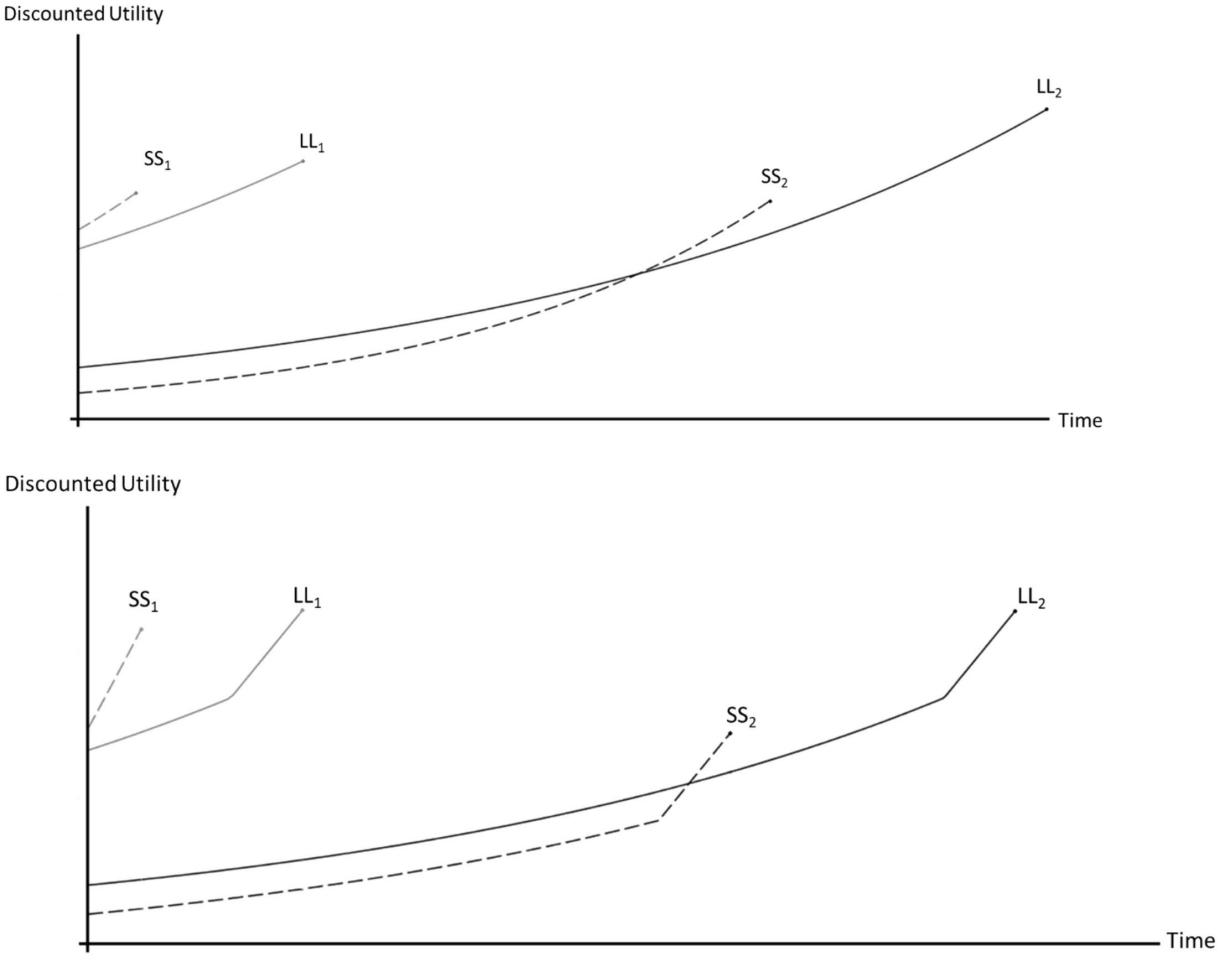
A preference reversal as predicted by hyperbolic discounting (top) and quasi-hyperbolic (bottom). In each panel, a decision maker has two intertemporal choices: “SS_1_ or LL_1_?,” and, “SS_2_ or LL_2_?” As can be seen by the respective intercepts on the discounted utility y-axis, SS_1_ if preferred to LL_1_ (due to having a higher level of discounted utility), and LL_2_ is preferred to SS_2_. Yet eventually time will pass, such that SS_2_ occupies the current position in time as SS_1_, and LL_2_ similarly for LL_1_. At this delay, the choice becomes identical to the choice between SS_1_ and LL_1_, meaning that SS_2_ will eventually be preferred to LL_2_. This pattern constitutes a preference reversal.

Figure 2 illustrates preference reversals using two forms of hyperbolic discounting. The top panel follows Loewenstein and Prelec’s (1992) traditional hyperbolic function, and the bottom panel follows Laibson’s (1997) quasi-hyperbolic function. In both panels in Figure 2, the first smaller-sooner reward (SS_1_) has a higher present value, and is therefore preferred, to the first larger-later reward (LL_1_). The second choice, between SS_2_ and LL_2_, is identical to the choice between SS_1_ and LL_1_, with both rewards being shifted by a constant amount into the future. Here, though, LL_2_ has a higher present value than SS_2_, meaning that LL_2_ is preferred at that longer delay. But these preferences are predicted to reverse once time has progressed such that SS_2_ and LL_2_ occupy the positions that SS_1_ and LL_1_ are currently in. This preference reversal occurs under both the hyperbolic and quasi-hyperbolic models; both models lead to the same behavioral prediction, despite differences in the shapes of their curves.

### 4.3 Two issues with the hyperbolic discounting model and other descriptive theories

The logic behind hyperbolic discounting dates back to Strotz (1955), before being developed by Ainslie (1975), Loewenstein and Prelec (1992), and Laibson (1997). Hyperbolic discounting models involve relatively small changes to the rational intertemporal choice model, while successfully producing predictions consistent with important intertemporal failings. However, hyperbolic discounting models nevertheless assume that decision-makers can effortlessly access a mental picture of future events, and tend to focus on precommitment as a way of helping guide people toward achieving their long-term goals.

Like the rational model (Williams, 1938), hyperbolic discounting assumes that people can effortlessly consider future utilities. Returning to the earlier example, even a skilled stock analyst cannot just know the future stream of dividends for each firm without extensive estimation. Every new event forces a costly re-evaluation. In real world contexts, with ordinary decision-makers, such calculations are implausible. The assumption that individuals always “know” their future utilities fails in a world characterized by uncertainty and change. This argument, that it is unrealistic to expect decision makers to always know what the future utilities are in a changing world, applies equally well to the hyperbolic discounting model.

A second issue is how to best help hyperbolic discounters to make choices that are in their long-term interests. Strotz (1955) and Ainslie (1975) both use the example of Odysseus from classical Greek literature to demonstrate the tactic of precommitment. Odysseus was a captain of a ship that had to sail past a group of mythical creatures called the Sirens. The Sirens sang a song so beautiful, that any sailors who heard the song would be impelled to sail toward the Sirens, thereby wrecking their ship on the rocks. Odysseus wanted both to hear this song and escape with his ship, and accomplished this feat by filling his soldiers’ ears with wax (so they could not hear the Sirens) and by tying himself to the mast, so that he could not take control of the ship at the crucial time. This precommitment strategy prevented a costly preference reversal of sailing toward the Sirens. Laibson (1997) by comparison uses the more commonplace scenario of saving for retirement. A person prone toward preference reversals might actually prefer that their retirement savings be placed in an account which is either costly or difficult to access. These costs or impediments can serve as a precommitment device to keep saving for retirement, to lessen the feasibility of the temptation to spend the money right away. Precommitment devices can be used successfully (Bryan et al., 2010). For example, the website stickk.com has helped over 600,000 people set up contracts specifying financial penalties if they fail to attain some desirable and difficult goal.^2^

Yet precommitment devices have drawbacks in a changing world: “Since these devices make future behavior less flexible, they may reduce rather than increase reward in a changing environment” (Ainslie, 1975, p.475). Strotz (1955, p.177) also wrote that “precommitment is only occasionally a feasible strategy (because of risk and uncertainty)”. It has already been argued that a changing world reduces the feasibility of forecasting all future utilities as required by either the rational intertemporal choice model or hyperbolic discounting. Given this observation, it would be useful for a rival intertemporal choice model to provide other advice on how to make better intertemporal choices given that the world will likely keep changing.

Other descriptive theories exist in the intertemporal choice field outside of hyperbolic discounting. Theories of logarithmic time perception argue that time perception is not linear, such that one unit of psychological time corresponds to a longer interval of objective time in the long- than short-term (Ventre and Martino, 2022; Zauberman et al., 2009). If people think in terms of logarithmic psychological time, this has been proposed as a mechanism underlying hyperbolic discounting (Ventre and Martino, 2022; Zauberman et al., 2009). Decision by sampling theory states that decisions are made based on the rank-ordering of different prospects, which could explain intertemporal choice failings if there are more relevant future utilities in the short- than long-term (Stewart et al., 2006). Temporal construal theory states that the short-term is seen in much richer and more specific detail than the long-term (Trope and Liberman, 2003), which could explain why intertemporal choice failings recur in the short-term. These are all valid aspects regarding the richness of intertemporal choices that the present model does not refute.

However, none of them provides a clear psychological strategy that someone could follow to try to act more consistently with their long-term wishes. This is the issue motivating the present mathematical model, which aims to show how regrettable intertemporal choice failings can also be avoided by expanding a finite window of attention into the future.

## 5 Mathematical model

Our model presents a simple framework demonstrating the implications of decision-makers who have to mentally-simulate the future during important naturalistic choices (Atance and O'Neill, 2001), and which is consistent with the four intertemporal biases described above.

### 5.1 Assumption 1

The first assumption of our model addresses a person’s perception of the future horizon, and how the utilities occurring before that point are discounted. To understand that, consider “Alice” who is constrained in how far forward she can extend her rational intertemporal preferences for any given choice. Alice has some stable lifetime utility function U(·), representing her welfare. Given that this is an intertemporal problem, this will be determined by the payoffs, *x_t_*, she receives at particular points in time, *t*. Alice also has some stable lifetime rational discount function, e^-rt^, which is used to discount utilities received in the future (*t* > 0). For the current purposes this is illustrated here in continuous time (Samuelson, 1937), although discrete time formulations would also be possible. An Alice with a high “discount rate” (r) puts a higher lifetime weight on present than future rewards, compared to an Alice with a lower r∈[0,∞) . But Alice’s bounded rationality means that she can only consider utilities that are realized until her “subjective attentional horizon” (S), which represents the last time period that she has been able to mentally simulate.^3^

In accordance with the rational intertemporal choice model (Samuelson, 1937), Alice *should* discount until her “objective time horizon,” (T), representing the last time period that she cares about:

$$\max_{x_{t}}U(.)=\int_{t=0}^{S}u(x_{t})e^{-\mathrm{rt}}\mathrm{dt}+\int_{t=S}^{T}u(x_{t})e^{-\mathrm{rt}}\;\mathrm{dt}$$ (1)

Where *S* ≤T,and T∈(S,∞) .

Equation 1 expands out the standard Samuelsonian integral between zero and T into two equivalent intervals: ∈(0,S] and ∈(S,T] . However, Alice is unable to account for any rewards beyond S (the second integral in Equation 1), giving them zero weight, even though they matter to her. We allow S to vary for each intertemporal choice, reflecting the need to flexibly reflect on novel decisions in a changing world. Our Assumption 1 therefore is that Alice’s choices can be described via Equation 2’s S-Maximization (which removes the second integral from Equation 1):

$$\max_{x_{i}}U(x)=\int_{t=0}^{S}u(x_{t})e^{-\mathrm{rt}}\mathrm{dt}$$ (2)

Alice therefore acts like Samuelson’s (1937) rational intertemporal agent when she has sufficient attentional resources to set S = T.

The assumption of a discontinuity at S is kept deliberately simple. It might appear that this assumption will lead to a bimodal prediction, where behavior is either extremely rational or irrational However, Assumption 1 is consistent with a smooth continuum of intertemporal choice failings for any source of utility which is spread out through time. For example, intertemporal preferences are often measured in the laboratory by asking participants to value a monetary reward at time *t*. It might seem that, in the present model, money received at some future delay should either be discounted entirely if this happens after the subjective attentional horizon (i.e., if t > S), or discounted by e^-rt^ otherwise. However, money has value to consumers only to the extent that it can support higher consumption, an activity which is itself spread over time (Cubitt and Read, 2007). The present model can therefore create a continuum of valuations for money, to the extent that consumption needs to be planned over S.

For example, suppose Alice has inherited $100,000, under the stipulation that the money can be spent at any time in the next 10 years (T = 10). Assume Alice has the utility function U(x) = x^0.5^, a standard diminishing marginal utility function meaning she benefits from spreading the spending out over the 10 years. However, an Alice with an S < 10 will “blow her inheritance” sooner than that. If Alice also has r = 0.05, then it is possible to show through Hamiltonian optimization (Ross, 2015), that she gains 8.1 times more utility from the inheritance by spending it over 10 years than by spending it over the next year. Utility losses occur for any diminishing marginal utility function, and converge to zero either as the subjective attentional horizon approaches the objective time horizon, or because high discount rates mean that it is only “rational” to consider the short-term anyway, as r - > infinity. Alice values this money received in the present smoothly, based on how far into the future she plans spending it. This perspective could help understand patterns of actual pension spending, where initial periods of overspending often result in the pension pot becoming exhausted (and thus consumption thereby plummeting) long before retirement ends (Newall and Peacey, 2021).

This same result holds true for *any* source of utility which is spread over time. Even experiences which occur in a short interval of time could be valued smoothly, to the extent that pleasurable memories contribute to the total utility of an experience (Loewenstein and Elster, 1992).

### 5.2 Assumption 2

The second assumption of our model specifies the relative difficulty of mentally simulating different parts of the future, first by defining the point at which mental costs begin, and second by explaining how these costs change as we project further into the future. First, we assume that attentional resources are only consumed from the first unique utility for one of the intertemporal options in a given choice, termed the “attentional starting point,” $\;t_{A}$ . This is because under the rational intertemporal choice model, which Alice follows until S, discounting until $t_{A}$ will always be equivalent to discounting until t = 0. For example, consider if Alice is deciding whether to move to the US in 1 year, or to move to Australia in 2 years. No matter Alice’s choice, her utilities for the first year will be identical, and are therefore irrelevant to her choice. The worth of ignoring irrelevant utilities can be argued for on the same grounds of bounded rationality—the efficient use of scarce cognitive resources.

Second, we further assume a diminishing attentional resource-requirement of extending S: That pushing the attentional starting point further into the future increases the total horizon beyond $t_{A}$ which can be considered for a given budget of attentional resources. In other words, it takes more cognitive resources for Alice to mentally simulate the next 24 h than for her to mentally simulate the same day 1 year in the future.

Therefore, Assumption 2’s components can be described mathematically by the following:

$$S(t_{A},R)=t_{A}+f(t_{A},R)$$ (3)

When

$$R>0,f(t_{A},R)>0,\frac{\partial f(t_{A},R)}{\partial t_{A}}>0,\frac{\partial f(t_{A},R)}{\partial R}>0,\frac{\partial f^{2}(t_{A},R)}{\partial t_{A}^{2}}>0.$$

Equation 3 states that the subjective attentional horizon (S) length beyond the attentional starting point $\left(t_{A}\right)$ depends on the cognitive resources available (R) and the attentional starting point. When positive resources are available ( R>0) , the restrictions on the function $f(t_{A},R)\;$ explain the specifics of how the two parameters influence S. First, any combination of $t_{A}$ or R will always increase S toward T $,(f(t_{A},R)>0)$ . Second, the restrictions placed on first order partial derivatives of *f* $\left(\frac{\partial f(t_{A},R)}{\partial t_{A}}>0\;\text{and}\;\;\frac{\partial f(t_{A},R)}{\partial R}>0\right)$ , require increases to the attentional starting point (or available resources) to increase S. Finally, the restriction placed on the second order partial derivative of *f* with respect to $t_{A}$ , $\left(\frac{\partial f^{2}(t_{A},R)}{\partial t_{A}^{2}}>0\right)$ , requires that increases to the attentional starting point have an increasing effect on S. This means that, for a constant level of R, the subjective attentional horizon increases more rapidly as the attentional starting point moves further into the future. If no resources are available, then the subjective attentional horizon is simply equal to the attentional starting point, $f(t_{A},R)=0$ . We note that this assumption is intended as a tractable formalization, and an important direction for future work is to test the assumption’s specific functional form.

A pension saving example can help to illustrate Assumption 2. With pensions, workers only gain benefits throughout their retirement by sacrificing their present spending power. This reduction in present spending power therefore acts as the attentional starting point for any immediate pension savings decision. But as the “Save more tomorrow” programme shows, workers will commit to higher pension contribution rates if those contributions come from future increases in income (Thaler and Benartzi, 2004). Shifting the consequences of the pension saving decision into the future shifts the attentional starting point further into the future, which can lead to more patient choices due to the diminishing attentional resource-requirement of extending S.

The present model does not attempt to explain why it is easier to think about the long-term than short-term, just as how hyperbolic discounting does not attempt to explain why discount rates decline into the future. Here we will point to three potentially complementary psychological theories for why it might be easier to think about the long- than short-term. Decision by sampling theory states that intertemporal choice failings follow when there are more relevant future utilities in the short- than long-term (Stewart et al., 2006). It could be easier to think about the long-term because there is less there that matters to us. Temporal construal theory states that the short-term is seen in much richer and more specific detail than the long-term (Trope and Liberman, 2003). It could be that a given utility is easier to think about in the long-term due to its higher-level representation there. Logarithmic time perception states that one unit of psychological time corresponds to a longer interval of objective time in the long- than short-term (Ventre and Martino, 2022; Zauberman et al., 2009). It could be that the long-term is easier to think about because an objective interval of long-term time feels shorter than an equivalent short-term interval. The special difficulty of dealing with the short-term may well be multiply determined, in a way which is beyond the explanatory scope of either hyperbolic discounting or the present model. The present framework is therefore consistent with these perspectives, but differs in using them to justify why a diminishing attentional resource-requirement of extending S is plausible, which then in turn generates the model’s concrete behavioral predictions.

### 5.3 Three errors

In this section we formally define all of the errors that can result from Assumption 1. In each case, the error occurs because of some change in utility beyond the last time period thought about.

Here we categorize Alice’s potential errors when choosing over any positive discrete number of intertemporal prospects. Utilities realized during ‘the void’ (V) between the subjective attentional horizon and the objective time horizon (i.e., between S and T) are incorrectly given zero weight, despite rationally contributing to discounted present value (T = S + V). We start by assuming there exists a status quo option, which is normalized to provide a flow of zero utility (‘the null’ {∅} ). With only a single non-null intertemporal option, Alice can make only two potential errors:

[B] “Bacon” has initial positive *“considered utility”* until S, but sufficient negative utility afterward in V that *“total utility”* until T is negative $\left(B_{S}>0>B_{T}\right)$ . Bacon has initial positive utility, but should not be chosen based on its long-run costs. Bacon is incorrectly chosen, given the choice set {B,∅} .

[C] “Carrots” have initial negative *“considered utility”* until S, but sufficient positive utility afterward in V that *“total utility”* until T is positive $\left(C_{T}>0>C_{S}\right)$ . Carrots have initial negative utility, and so will not be chosen despite their long-run benefits. Carrots are incorrectly rejected, given the choice set {C,∅} .

These first two errors are not dependent on the null; they still occur if the null is replaced by some other non-null option, D, as long as a cross-over in present values occurs between S and T. In error [B] this occurs when Bacon is initially better, $B_{S}>D_{S}\;$ ; but is worse in the long-run $D_{T}>B_{T}$ . In error [C] this occurs when Carrots are initially worse, $D_{S}>C_{S}\;$ ; but better in the long-run $C_{T}>D_{T}$ . We require no other assumptions on the utility stream of D for errors [B] and [C] to occur [D could be globally non-negative, or have negative cumulative present value at some point(s)].

There is one additional potential error, which does not involve *any* present values crossing the zero line:

[B-C] “Bacon” has an initial *“considered utility”* until S which is greater than “Carrots” $\left(B_{S}>C_{S}\geq 0\right)$ . However, the *“total utility”* until T is greater for Carrots than Bacon $\left(C_{T}>B_{T}\geq 0\right)$ . Bacon is chosen, given the choice set {B,C,∅} , when Carrots would have ultimately been better.

**Figure 3:**
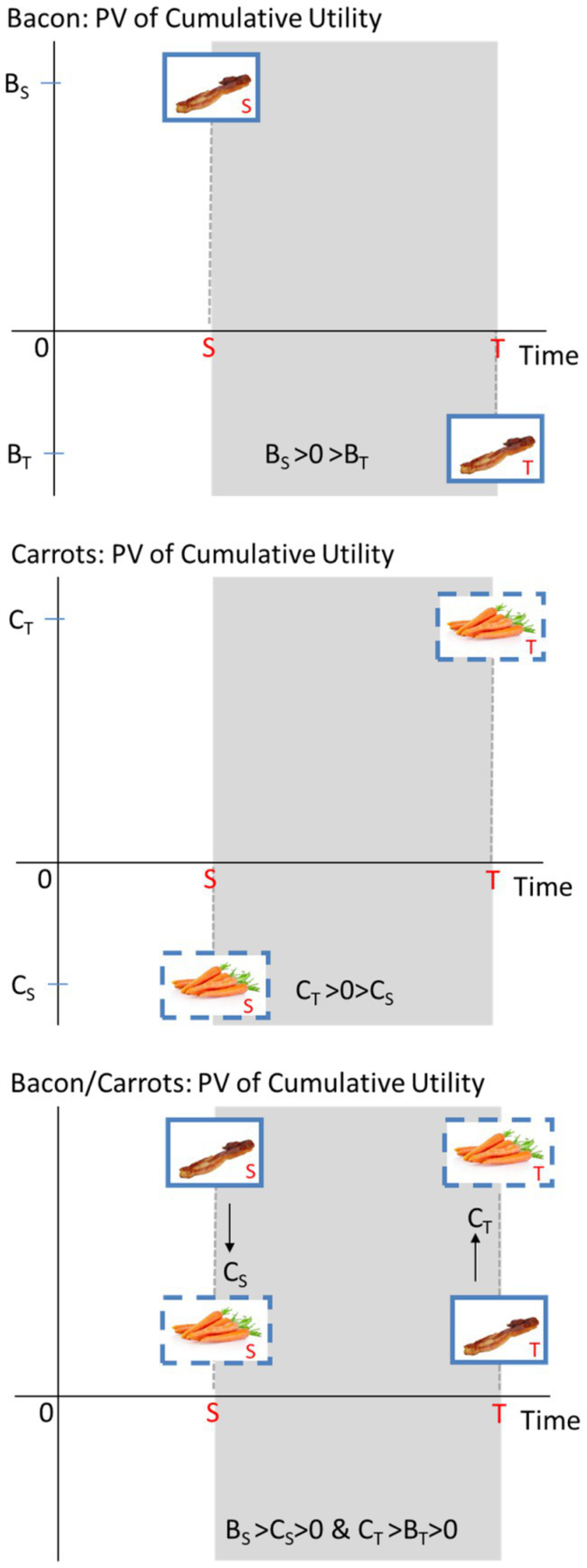
Categorization of errors. Alice mistakenly chooses Bacon, despite its long-term costs [B]. Alice mistakenly rejects Carrots, despite their long-term benefits [C]. Alice mistakenly chooses Bacon, when Carrots would have ultimately been better [B-C]. All three errors occur because the subjective attentional horizon (S) is before the objective time horizon (T).

All three errors are illustrated in Figure 3.

We believe that a direct correspondence can be made between the important intertemporal biases i–iii. Described above and the three possible errors following from Assumption 1. Addictions have high initial rewards but ultimately reduce intertemporal welfare compared to abstinence (Orford, 2001). The corresponding error is Bacon in Error [B]. Procrastination tends to happen with initially aversive activities, but where delay ultimately reduces intertemporal welfare compared to immediate action (Akerlof, 1991; Steel, 2007). The corresponding error is Carrots in Error [C]. The smaller-sooner bias involves taking something now, rather than something sufficiently larger, later. If the loss of later utility is sufficiently large, this can be an error even if the smaller-sooner reward itself has positive utility. The corresponding error is Bacon-Carrots in Error [B-C].

### 5.4 A fourth error

**Figure 4:**
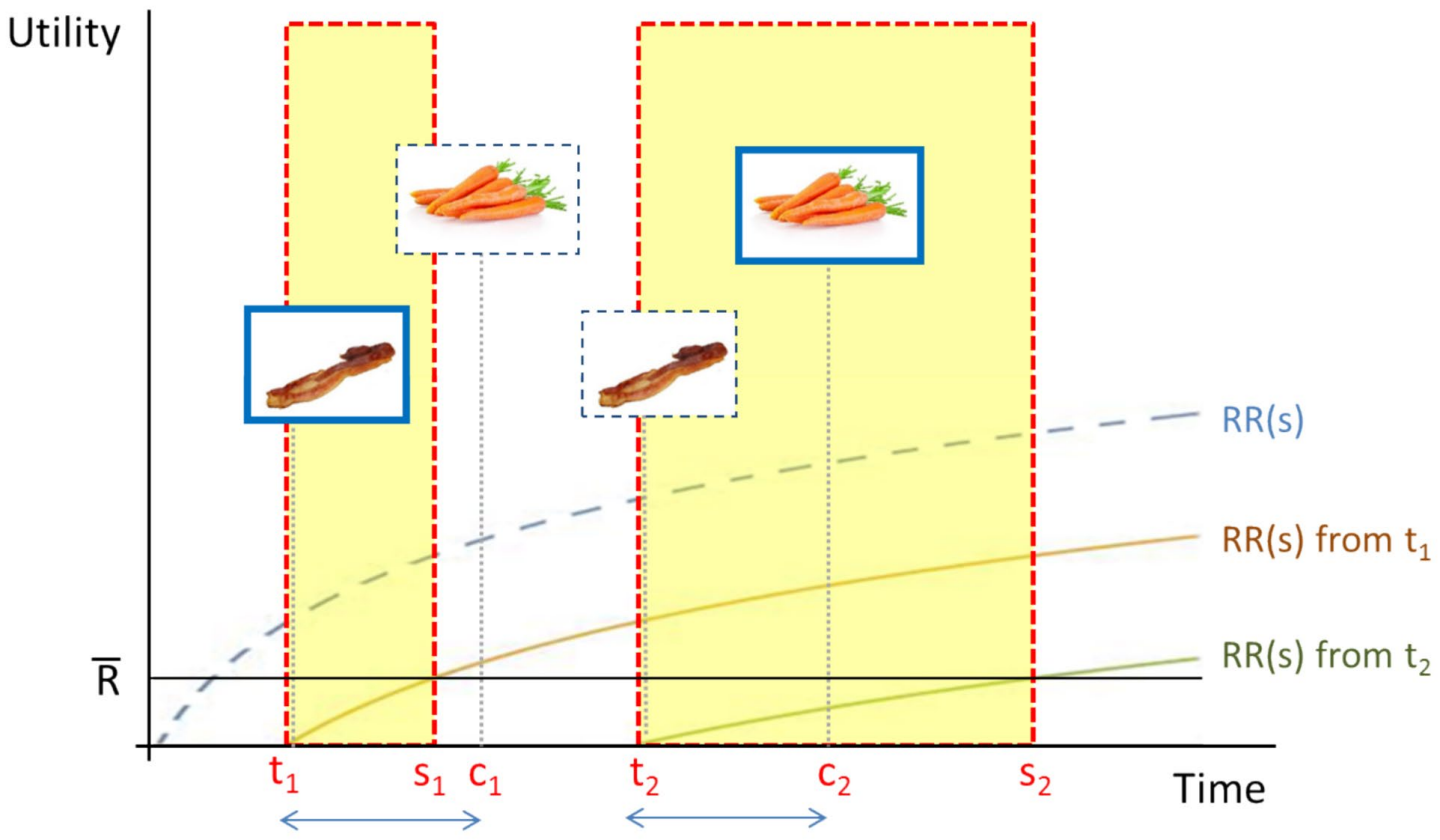
A diminishing attentional resource-requirement of extending the subjective attentional horizon (S), RR(s), beyond the first unique utility (t_1_ for the first choice, and t_2_ for the second choice). Solid square boxes illustrate the chosen option in both choices. An equal budget of cognitive resources $\left(\bar{R}\right)$ is used to extend S in both choices. In the first choice, some of the positive utility of Carrots (c_1_) lies beyond S_1_, and so Alice chooses Bacon, the positive utility of which is all within S_1_. In the second choice, the total utility of Carrots (c_2_) occurs before S_2_, and so now Alice’s choice correctly switches from Bacon. The vertical axis corresponds to utility in terms of the rewards from consumption (of bacon or carrots) and the resource costs of extending S from the attentional starting point (t_1_ or t_2_).

The biases i.-iii. Can appear obviously welfare-reducing when seen at a distance, but become much more tempting as the first relevant utility creeps closer to the present. These are preference reversals (bias iv. above), and are well-known to groups including addicts trying to stay clean, students trying to complete assignments, or workers trying to save for the future. Our Assumption 2 means that errors [B], [C], and [B-C], and only those errors, are more likely (although not guaranteed) to happen as a given intertemporal choice is evaluated at a shorter delay. A combination of Assumption 1 and Assumption 2 is therefore consistent with bias iv. Figure 4 illustrates this mechanism via a Bacon-Carrots choice.

These predicted preference reversals mean that Alice could benefit from precommitment devices, like in hyperbolic discounting (Ainslie, 1975; Laibson, 1997; Strotz, 1955). However, hyperbolic discounting makes deterministic predictions about whether a preference reversal will occur in any given situation. A hyperbolic discounter is essentially guaranteed, absent external help, to make a preference reversal in any given situation, due to their discount function. By contrast, the present model predicts that an individual can also help herself to make more patient choices in any given situation, by thinking further into the future. As argued next, this appears consistent with the success of a range of different intertemporal-choice interventions.

## 6 Interventions on naturalistic intertemporal choices

In this section we review studies that manipulate some aspect of participants’ future thinking and measure an intertemporal choice as an outcome. Given the present model’s intended domain, we will review studies that measure naturalistic outcomes or that are as close as possible to naturalistic outcomes in a given literature.

A literature has recently emerged suggesting that episodic future thinking, the imagination of personal future events, has beneficial effects on a range of intertemporal failings. Initial studies explored mainly episodic future thinking’s effects on choices among laboratory-based monetary rewards (Rung and Madden, 2018). However, this literature is now progressing to measure laboratory-based versions of intertemporal choice problems that are more commonly faced in naturalistic domains, and on populations that particularly struggle with them. A meta-analysis has recently been conducted in this area (Rösch et al., 2022), suggesting that the intervention has as large a beneficial effect on improving health-related choices, such as overeating (Daniel et al., 2013), as it does on choices among monetary rewards. One study in particular showed a significant reduction in calorie consumption in a food court, which is a naturalistic outcome (O'Neill et al., 2016). A recent systematic review has suggested beneficial effects on people with substance-based issues (Collado and Stokes, 2024). Although naturalistic choices are harder to measure among this group, the current evidence base suggests beneficial laboratory-based outcomes on cigarette smoking (Stein et al., 2016), and laboratory-measured levels of alcohol consumption (Athamneh et al., 2022).

Hershfield et al. (2011) tested the influence of age-progressed renderings of participants’ faces on investment behavior. Participants exposed to age-progressed renderings of their own face took significantly higher care for future financial outcomes than either participants seeing their present face, or participants seeing another age-progressed face (Hershfield et al., 2011). Furthermore, a related field study using 50,000 people saving for retirement found that this age-progression intervention led to a significant increase in the number of people making one-time pension contributions – a relevant naturalistic outcome (Robalino et al., 2023). A randomized-controlled-trial in Turkish schools, which aimed to help children imagine the future consequences of their actions, found that children acted more patiently in incentivized tasks up to 3 years after program completion (Alan and Ertac, 2018).

## 7 Testing the model

The testing of unique predictions is a way to produce evidence for or against any scientific perspective. One reason for the present work’s theoretical focus is that we, the authors, do not have access to sufficient research resources to test the model in a way that readers are likely to find convincing. Naturalistic intertemporal choices are this model’s intended explanatory domain, which should have a greater need for mental simulation than experimental situations. We therefore in this section propose some ways in which the model’s unique perspectives could be subjected to independent empirical testing.

Although the episodic future thinking literature provides robust guidelines on how to best manipulate future thinking (Hollis-Hansen et al., 2019), the best study here would be one which objectively records naturalistic outcomes via a randomized controlled trial. While a randomized future thinking intervention can be implemented via smartphone-based apps (O'Neill et al., 2016), it is harder to objectively record choice outcomes, particularly if a longitudinal study wanted to observe whether this intervention works over long time periods across multiple environments. Psychology has recently followed physics in terms of testing theories via large consortia of collaborative scientists (e.g., Klein et al., 2018; Lucca et al., 2025), and so this could be a problem best tackled in a similar way.

Compared to hyperbolic discounting, the present model implies a higher potential worst-case scenario for intertemporal choice errors. In the present model, any arbitrarily high reward beyond the subjective attentional horizon (S) will be ignored. This contrasts with hyperbolic models, where the intertemporal choice failing parameter(s) specify the maximum trade-off across time (Ainslie, 1975; Laibson, 1997; Loewenstein and Prelec, 1992; Strotz, 1955). The current global lack of action on climate change and plastic pollution appear to reflect an essentially-total population level disregard for events beyond a fairly immediate horizon. While large group problems such as these can be exacerbated by collective action problems, this is less so for addiction, where the welfare costs are borne by much smaller groups (Orford et al., 2013). For example, the observation of smokers with cancer diagnoses who continue to smoke despite severe pain and their overall health condition, suggests that people suffering from addiction can continue to pursue short-term rewards even in the face of overwhelming consequences (Daniel et al., 2009). Research could therefore explore whether some naturalistic choices neglect future consequences to such a degree as to effectively rule out hyperbolic discounting as an explanatory theory. This has been done in risky choice, where the rejection of small-stakes gambles has been shown to effectively rule out risk aversion as a descriptive theory in favor of loss aversion (Rabin, 2000).

Another route for empirical testing lies in the second assumption of diminishing attentional resource-requirement. That is, a given time interval such as 24 h requires fewer attentional resources to be simulated, the further in the future that this interval exists. Certainly, a grandparent will have more to think about in the next 24 h, than they would for a given 24 h period when they forecast that only their family and not they themselves will still be alive. However, this assumption would benefit from direct empirical testing.

Unfortunately, we are unable at this time to work out testable differences between this perspective and intertemporal choice models beyond hyperbolic discounting. These models are numerous (Ballard et al., 2023), and have been developed over time to explain a number of effects in laboratory-based choices among monetary rewards (Rambaud et al., 2023), which we argue to be beyond the scope of the present work.

## 8 Conclusion

Naturalistic intertemporal choices often display the important biases of addiction, procrastination, choosing smaller-sooner over larger-later rewards, and caving into these choices in the present despite a desire to avoid them in the future. These everyday biases have been known about at least since ancient Greek times (Ainslie, 2001), and collectively reduce societal welfare, which can be seen perhaps most clearly by addiction’s harms on those who are afflicted and by their families (Orford et al., 2013). While the established theory of hyperbolic discounting can explain simple laboratory-based choices in animals and humans which capture these essential dynamics (Ainslie and Haslam, 1992), more complex scenarios have led to a kaleidoscope of findings (Rambaud et al., 2023) and theories (Ballard et al., 2023). Contrastingly, our focus here is on the naturalistic intertemporal errors described above, which we argue can be understood by the need to mentally simulate the future in our changeable world. The model that follows from our two assumptions is consistent with these four errors, and predicts that decision-makers can mitigate their welfare impacts by thinking further into the future. This work is intended to stimulate empirical testing and debate in the field, to best consider ways that people can be helped to achieve their long-term aims. A comparison between the present model and those of exponential and hyperbolic discounting are shown in Table 1.

**Table 1:** Comparison of three intertemporal choice models.

Model feature	Exponential	Hyperbolic	Present model (S, T)
Indifference curve shape	Linear in log(time)	Convex	Linear in log(time) until kink at S
Preference reversals	None	Always a present bias	Occurs if and only if critical option is before S
Cognitive load	No effect	No effect	Reduces S, which is likely to increase intertemporal choice failings
Policy lever	N/A	Precommitment devices	Manipulate episodic future thinking

The present model leaves many open issues for future research. The model does not attempt to explain cases of excessive patience (Loewenstein, 2018), an intertemporal bias that is arguably less widespread than those described here. Potential adaptive reasons for not thinking about the future, such as an avoidance of the fear of death (Becker, 1973), were not considered. Our treatment of preference reversals does not relate to situations where additional information makes it rational to change one’s mind (McGuire and Kable, 2013). As in the rational intertemporal choice theory and hyperbolic discounting, the present theory also assumes that all future utilities can be correctly anticipated. This assumption is likely to be less accurate for decisions makers experiencing extreme emotions, or for people subject to certain dispositional traits (Bulley et al., 2016). Introducing a bias term for future utilities could plausibly account for these additional factors. Model complexity could also be increased by for example allowing some future utilities to be weighted partially. However, a strength of the present theory lies in its relatively simple set of alterations to the rational intertemporal choice model. We also leave open issues regarding whether thinking about the future is a skill that people can develop with practice, and call for the field to consider ways in which these perspectives might be tested convincingly.

The world keeps changing, and yet we can always look beyond the present and toward a better future.

It will be worth it, in the end.

## Data Availability

The original contributions presented in the study are included in the article/supplementary material, further inquiries can be directed to the corresponding author.

## Footnotes

1 These choices were repeated a sufficient number of times so that the animal could learn these rewards with certainty (Herrnstein, 1997).

2
https://stickk.zendesk.com/hc/en-us/articles/206109308-What-is-stickK

3 Future work could explore a time horizon which is probabilistically-defined, so that utilities beyond S have some non-zero probability of being accounted for.
